# Case report: Cerebrotendinous Xanthomatosis masquerading as adult ADHD in psychiatric practice

**DOI:** 10.3389/fpsyt.2025.1528204

**Published:** 2025-02-04

**Authors:** Jongtae Kim, Yun Jeong Hong, Si Baek Lee, Seong Hoon Kim, Myung Ah Lee, Eunbuel Ko, Jeong Wook Park

**Affiliations:** ^1^ Department of Psychiatry, Uijeongbu St. Mary’s Hospital, The Catholic University of Korea, Seoul, Republic of Korea; ^2^ Department of Neurology, Uijeongbu St. Mary’s Hospital, The Catholic University of Korea, Seoul, Republic of Korea

**Keywords:** Cerebrotendinous Xanthomatosis, Attention-Deficit/Hyperactivity Disorder, case report, differential diagnosis, neuropsychiatry

## Abstract

**Introduction:**

This case report details the presentation of a patient who initially sought consultation at a psychiatric outpatient clinic with symptoms suggestive of Attention-Deficit/Hyperactivity Disorder (ADHD); however, further evaluation revealed a diagnosis of Cerebrotendinous Xanthomatosis (CTX). CTX, a genetic disorder impacting lipid metabolism, is often overlooked in differential diagnoses due to its rarity. This case underscores the importance of considering alternative diagnoses in adults exhibiting ADHD-like symptoms without a childhood history of the disorder, adding to the literature on diagnostic complexities in psychiatric practice.

**Case presentation:**

A 33-year-old man visited a psychiatric outpatient clinic with symptoms such as increasing distractibility and inattention, requesting an evaluation for adult ADHD. However, the absence of an ADHD history in childhood, coupled with progressive neurological symptoms and tendon xanthomas, led to further investigation. Comprehensive neurological assessments, including neuroimaging and genetic testing, ultimately diagnosed him with CTX. Treatment with chenodeoxycholic acid (CDCA) resulted in stabilization of cognitive function, although improvement in gait disturbances and tremors remained minimal.

**Conclusion:**

This case demonstrates that CTX can masquerade as adult ADHD, emphasizing the necessity for thorough assessments in atypical ADHD presentations. Psychiatrists should consider rare metabolic disorders like CTX in similar cases, which may enable timely intervention and improve patient outcomes.

## Introduction

Studies have found a significant increase in Attention-Deficit/Hyperactivity Disorder (ADHD) diagnoses and medication use among Korean adults, with the prevalence of diagnosed ADHD rising tenfold from 2008 to 2018 (1, 2). In clinical practice, adults presenting with ADHD symptoms often reveal, upon closer examination, a recent onset of these symptoms with no corresponding childhood history, suggesting an alternative etiology (3). Therefore, a comprehensive differential diagnosis is crucial in ADHD assessment to exclude other potential causes that may mimic ADHD symptomatology.

Cerebrotendinous Xanthomatosis (CTX) is a rare genetic disorder due to variants in the *CYP27A1* gene, inherited in an autosomal recessive manner (4). CTX affects lipid storage and is associated with abnormalities in bile acid biosynthesis pathways (4). It causes the buildup of cholesterol metabolites and other harmful intermediates, such as cholestanol, in tissues, especially in the brain, eye lenses, and tendons (5). Accumulation of harmful intermediates is thought to result in the symptoms observed in CTX, including progressive neurological issues, cataracts, osteoporosis, diarrhea, cardiovascular disease, and xanthomas (5). Individuals with CTX often present neuropsychiatric symptoms, including cognitive impairment and behavioral disturbances (5). Therefore, children or adolescents showing these signs should be further evaluated for a possible CTX diagnosis, as early initiation of treatment can substantially mitigate the disease burden (5, 6).

This paper presents the case of a 33-year-old man who initially reported symptoms of ADHD but was later diagnosed with CTX. This case is unique due to its presentation of CTX mimicking symptoms commonly associated with adult ADHD, highlighting a critical diagnostic challenge in psychiatric practice.

## Case presentation

The patient, a 33-year-old man with an associate college degree, presented to a university-affiliated psychiatric outpatient clinic for assessment due to increasing issues with distraction, attention deficits, and impaired judgment over the past 3-4 years, impacting his ability to maintain employment. He had been referred from a local psychiatric clinic with a suspected diagnosis of adult ADHD. His medical history included a diagnosis of Graves’ disease five years prior, treated with methimazole. He had no family history of neurological disorders and no record of seizures or other significant health issues.

During the psychiatric evaluation, an extensive history obtained from both the patient and his parents indicated a lifelong tendency toward mild inattention and organizational difficulties, though these issues had not previously affected his overall functioning until recently. Over the past 2–3 years, he reported experiencing mild lethargy, itchy skin, frequent misplacement of personal items, and psychomotor agitation. His Full-Scale IQ (FSIQ) score on the Korean version of the Wechsler Adult Intelligence Scale-Fourth Edition (K-WAIS-IV) was 65, suggesting cognitive abilities within the range typically associated with mild intellectual disability. He also met four out of six criteria on the Adult ADHD Self-Report Scale Version 1.1 (ASRS-v1.1) Screener, suggesting the presence of symptoms consistent with ADHD (7).

Several factors complicated the diagnostic process. While lifelong mild inattention supported the possibility of adult ADHD, and the ASRS-v1.1 results reinforced this, premature diagnostic conclusions could be misleading. His history of Graves’ disease raised the possibility that thyroid dysfunction might contribute to his cognitive symptoms. Although his primary concern was ADHD, the recent pattern of cognitive deterioration was not a typical symptom of ADHD progression, warranting further investigation into other potential causes or co-occurring conditions.

**Figure 1:**
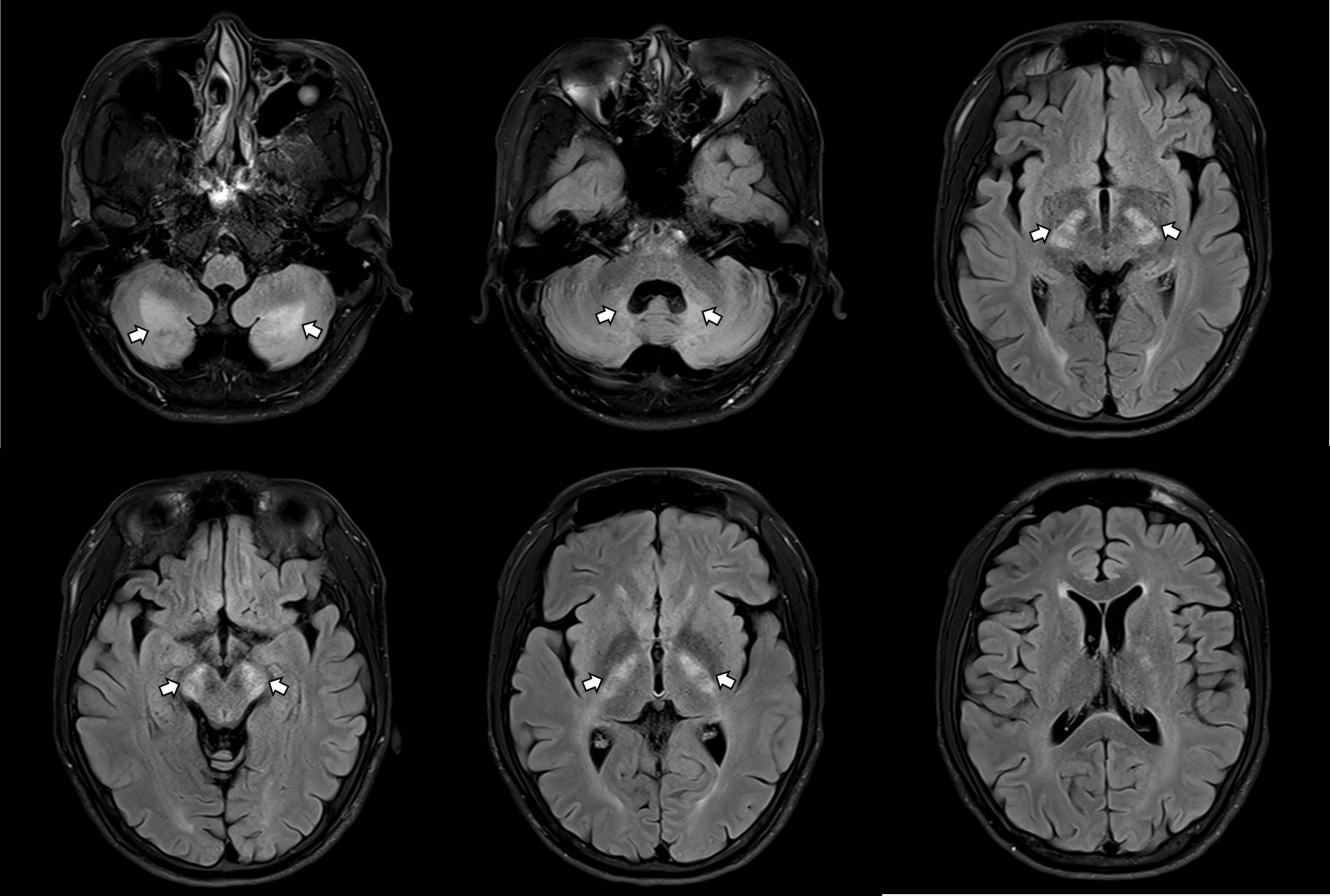
Brain MRI revealing symmetrical abnormalities in multiple brain regions.

The patient was referred to neurology for further assessment. On initial neurological examination, no definite gait abnormalities/ataxia/parkinsonism were observed, although he reported mild gait instability. He showed generalized hyperactive deep tendon reflexes in all extremities with bilateral positive Babinski signs. The initial Mini-Mental State Examination (MMSE) score was 28. Epileptiform discharges on EEG were not present, and nerve conduction studies (NCS) were unremarkable. Brain MRI revealed symmetrical high signal intensities on T2-weighted and fluid-attenuated inversion recovery (FLAIR) images in the posterior limb of the internal capsule, hypothalamus, cerebral peduncles, and cerebellar white matter, including the dentate nucleus (**Figure 1**). Because serum antithyroid antibody, anti-microsomal antibody, and thyroglobulin antibody were increased, Hashimoto’s encephalopathy could also be considered. Cerebrospinal fluid (CSF) analysis was performed to check for autoimmune diseases, infectious/inflammatory conditions, or malignancy, but the results were unremarkable. Steroid pulse therapy was ineffective.

**Figure 2:**
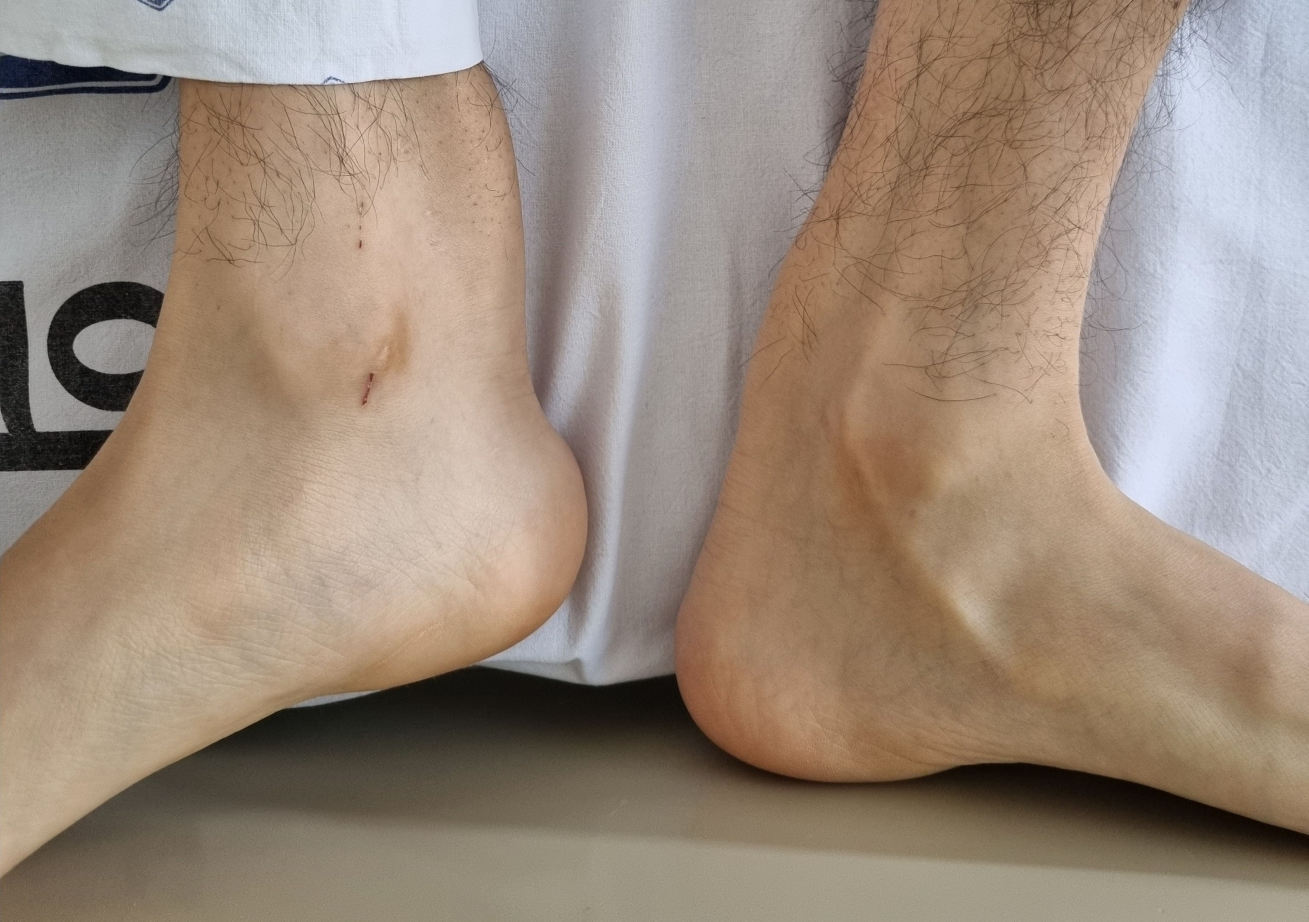
Bilateral Achilles tendon xanthomas.

About three months later, the patient reported an exacerbation of cognitive declines and gait disturbances, characterized by spastic gait and foot-dragging, which was accompanied by tremors in both legs. Physical examination revealed bilateral Achilles tendon xanthomas (**Figure 2**), presenting as painless, soft tissue masses. The follow-up MMSE score was 22, and detailed neuropsychological tests indicated amnestic multiple domain mild cognitive impairment (MCI) showing multiple cognitive impairments, including learning, memory delayed recall, frontal executive functions, and language function. Bilateral cataracts and osteoporosis were diagnosed concurrent with neurological deterioration, and the patient reported intermittent diarrhea. CTX was suspected based on brain MRI findings, xanthomas, and clinical presentations. A next-generation sequencing (NGS) genetic analysis was conducted to distinguish among various lipid metabolic disorders, including CTX. In genetic analysis, heterozygous pathogenic variants (c.1214G>A, c.1421G>A) in the *CYP27A1* gene, classified as pathogenic according to the American College of Medical Genetics and Genomics (ACMG) criteria (8), were identified, confirming CTX.

Treatment began with chenodeoxycholic acid (CDCA), the established standard of care for patients with CTX (5), at a dose of 250 mg three times daily, along with calcium supplements and cholesterol-lowering medication. The patient initially experienced mild nausea following medication initiation but subsequently tolerated the treatment well without further reported side effects. Medication adherence was assessed during outpatient visits and follow-up telephone checks, indicating high compliance. In response to the progression of ataxia, rehabilitation therapy was also initiated. After three months of CDCA therapy, although gait disturbances and tremors remained, the patient’s MMSE score improved to 30, and there was a noted reduction in both skin itching and intermittent diarrhea.

Important prognostic factors for this patient include the age at diagnosis, timing of treatment initiation, and characteristic CTX-related brain MRI findings (5). These indicators suggest a less favorable prognosis, as the patient’s MRI displayed typical CTX abnormalities, and the diagnosis was delayed until his mid-30s, by which point neurological symptoms had already developed. **Table 1** illustrates a timeline of the diagnostic process.

**Table 1:** Diagnostic timeline for Cerebrotendinous Xanthomatosis.

Date	Phase	Description
July 21, 2023	Initial Presentation	- Referred to a university-affiliated psychiatric outpatient clinic for evaluation of potential ADHD - Reported increasing difficulties with distraction, attention deficits, and compromised judgment
August 8, 2023	Psychiatric Assessment	- FSIQ assessed using the K-WAIS-IV: 65 - Met 4 of 6 criteria on the ASRS-v1.1 Screener - Late onset of ADHD-like symptoms warranted a thorough evaluation
August 18, 2023~	Neurology Referral	- Initial MMSE score: 28 - Brain MRI: Diffuse symmetrical high signal intensities on T2-weighted and FLAIR images, including the dentate nucleus - Elevated serum levels of antithyroid, anti-microsomal, and thyroglobulin antibodies - Hashimoto’s encephalopathy was considered as a potential diagnosis - Steroid pulse therapy showed no effectiveness
December, 2023~	New symptoms & Reevaluation	- Reported worsening cognitive decline and gait disturbance - Physical examination identified bilateral Achilles tendon xanthomas - Follow-up MMSE score: 22 - Bilateral cataracts and osteoporosis were diagnosed - NGS genetic analysis identified heterozygous pathogenic variants (c.1214G>A, c.1421G>A) in the *CYP27A1* gene, confirming CTX
June, 2024~	Treatment Initiation	- Initiated CDCA, calcium supplements, cholesterol-lowering medication, and rehabilitation therapy
October, 2024	Follow-Up	- Follow-up MMSE score: 30 - Gait disturbance and tremor persisted

ADHD, Attention-Deficit/Hyperactivity Disorder; FSIQ, Full-Scale IQ; K-WAIS-IV, Korean version of the Wechsler Adult Intelligence Scale-Fourth Edition; ASRS-v1.1, Adult ADHD Self-Report Scale Version 1.1; MMSE, Mini-Mental State Examination; FLAIR, Fluid-Attenuated Inversion Recovery; NGS, Next-Generation Sequencing; CTX, Cerebrotendinous Xanthomatosis; CDCA, Chenodeoxycholic Acid.

## Discussion

This case report highlights the complex diagnostic challenges faced in adult psychiatry. Although a single case cannot provide definitive generalizations for all patients with similar symptoms, and the diagnostic trajectory described here may differ from routine clinical experiences, the case offers meaningful insights for psychiatric assessment. The patient visited the hospital with a referral for suspected adult ADHD. However, the atypical late onset of severe symptoms and the progressive nature of his condition prompted a more comprehensive evaluation. This thorough approach, including neuroimaging and genetic testing, ultimately led to the diagnosis of CTX, a rare genetic disorder affecting lipid metabolism. This case illustrates the essential role of maintaining an expansive differential diagnosis and conducting in-depth evaluations when confronted with atypical psychiatric presentations.

CTX is diagnosed by the presence of biallelic pathogenic variants in the *CYP27A1* gene (5). The impaired activity of the CYP27A1 enzyme compromises the formation of CDCA and cholic acid, causing pathologic accumulation of cholesterol metabolites in tissues (5). Clinical presentations are highly heterogeneous, including juvenile cataracts (82%), tendon xanthomas (76%), cognitive impairments (87%), spasticity and pyramidal signs (64-92%), osteoporosis (>65%), chronic diarrhea (31%), and psychiatric disturbances (11.4%) (5). Regarding the variants, c.1214G>A missence variant is known to be related to spinal CTX or non-neurological CTX phenotype, while c.1421G>A missence variant is predicted to have classical CTX presentations (5). Our patient first presented with cognitive declines and psychiatric problems, and physical/neurological signs appeared later. Spasticity, gait instability, both leg tremors, cataracts, osteoporosis, and diarrhea were considered to be caused by CTX. Additionally, the patient also reported undiagnosed skin itching. Because pruritus is not widely described in CTX, we cannot definitively conclude whether it is directly related to the disease. Phenotypic variability, despite identical variants, may be influenced by environmental factors, unidentified genetic modifiers, or epigenetic influences (4).

Although a few symptoms, including itching, diarrhea, and cognitive declines, have improved after the CDCA medication, gait disturbance persists and limits his independence. Previous studies have reported that the age at diagnosis and initiation of CDCA treatment affect the clinical prognosis of CTX patients (5). Particularly, the presence of significant neurological deficits in CTX patients 25 years of age or older was associated with a poor prognosis (9). This case emphasizes the importance of comprehensive history-taking when evaluating adults with ADHD-like symptoms, as these may mask underlying conditions like CTX, delaying diagnosis. Adults with ADHD often struggle with organization, planning, and decision-making, leading to unstable jobs and relationships, as seen in the patient’s history (10). However, the recent exacerbation of his symptoms in adulthood raised concerns. Generally, symptoms appearing after age 13 suggest a diagnosis other than ADHD, which typically begins in childhood (11). Although the patient had a lifelong history of mild inattention, the recent significant cognitive decline and symptoms aggravation deviate from typical ADHD patterns. This clinical history has provided crucial insight for establishing a differential diagnosis. Therefore, clinicians diagnosing adult ADHD should thoroughly review childhood and adolescent histories for signs of inattentive or hyperactive-impulsive symptoms before age 12 to prevent misdiagnosis of other important conditions.

In addition, this case emphasizes the importance of thorough neurological workup in cases with atypical psychiatric symptoms. The initial psychiatric assessment suggested the possibility of organic causes other than ADHD, leading to a series of neurological diagnostic procedures, including a brain MRI and genetic studies. Most individuals with CTX show abnormalities on brain MRI scans (12). In the patient’s case, the MRI revealed abnormalities with high signal intensities on T2-weighted and FLAIR images, particularly in the dentate nucleus, a characteristic indicator of CTX (5). Alongside MRI, genetic testing of the *CYP27A1* gene is recommended for all patients suspected of having CTX (5). Following the recommendation, the patient received an accurate diagnosis of CTX via NGS genetic analysis. A limitation of this case was the lack of biochemical testing—such as plasma cholestanol or plasma/urine bile alcohol assays—due to unavailability during the evaluation. This case underscores the importance of collaboration between psychiatric and neurological specialists in managing complex cases and demonstrates the value of advanced diagnostic tools in identifying the causes of atypical neuropsychiatric presentations.

Identifying rare neurological disorders presenting with psychiatric symptoms, such as CTX, is particularly challenging for psychiatrists. The prognosis in CTX significantly improves with early diagnosis and prompt treatment initiation (5). However, the substantial heterogeneity in clinical presentations often leads to considerable diagnostic delays (5). At the time of the psychiatric unit visit, the patient presented with clinical features characteristic of CTX, such as cognitive impairment and tendon xanthomas. However, it is quite challenging for psychiatrists to accurately identify these manifestations and link them to rare neurometabolic disorders. In this case, the timely diagnosis of a rare condition was facilitated by the prompt collaboration with the neurology department. Considering that CTX-related symptoms in adults encompass psychiatric disturbances (5), it is conceivable that individuals with CTX may present to psychiatric units for evaluation and treatment. Thus, psychiatrists need to possess a comprehensive understanding of the distinctive clinical features of rare diseases that manifest psychiatric symptoms to enable timely diagnosis through interdisciplinary collaboration.

Despite an increase in the diagnosis and treatment of adult ADHD in Korea, the prevalence rates remain significantly lower compared to other countries (1). This disparity suggests that mental health professionals need to be more vigilant in considering ADHD as a potential diagnosis. However, this case report advocates for caution against prematurely diagnosing ADHD based solely on ADHD-like symptoms without a thorough evaluation. It emphasizes the importance of a comprehensive assessment to identify other significant conditions that may present similarly but require different interventions. By illustrating the value of interdisciplinary collaboration, the report highlights the critical balance between improving ADHD detection rates and avoiding misdiagnosis of other significant conditions. Thus, psychiatrists should be proactive in identifying adult ADHD while rigorously adhering to diagnostic standards to ensure accurate diagnosis and appropriate care.

## Patient perspective

“During the past few years, I noticed I was having increasing trouble focusing and keeping track of things, which made it challenging to keep a job. At first, I thought it might be ADHD since I’ve always had some minor attention problems. The cognitive issues were really frustrating - I kept losing things and couldn’t concentrate like I used to. When the physical symptoms started, especially the difficulty walking and tremors in my legs, I became more worried that something serious was wrong.

After starting the CDCA treatment, I’ve noticed some improvements. My skin doesn’t itch as much anymore, and the diarrhea has gotten better. My thinking seems clearer, too, though I still have trouble walking properly. While I wish I had been diagnosed earlier, I’m relieved to understand what was causing my symptoms finally and to be receiving proper treatment. The support from both the psychiatric and neurology teams has been helpful in managing my condition.”

## Data Availability

The original contributions presented in the study are included in the article/Supplementary Material. Further inquiries can be directed to the corresponding author.
